# Diabetes Self-Management Education in South Auckland, New Zealand, 2007-2008

**Published:** 2011-02-15

**Authors:** Martha Silva, Janet Clinton, Sarah Appleton, Pat Flanagan

**Affiliations:** University of Auckland, Auckland, New Zealand; Health Systems, School of Population Health, University of Auckland; University of Auckland, Auckland, New Zealand; Counties Manukau District Health Board, Manukau City, New Zealand

## Abstract

**Introduction:**

Self-management education programs seek to help patients realize that they are their own principal caregivers and that health care professionals are consultants who support them in this role. The aim of this study was to evaluate a diabetes self-management education program implemented as part of a district-wide approach in South Auckland, New Zealand, which has some of the highest prevalence rates for diabetes and is one of the most ethnically diverse and deprived regions of New Zealand.

**Methods:**

Self-management attitudes and behaviors were monitored with the use of questionnaires before and after program implementation. Clinical outcomes such as hemoglobin A1c, body mass index, and blood pressure were also tracked before the program began and 3 months after the program ended. Participant focus groups and facilitator interviews were conducted to explore perceptions of the program.

**Results:**

Participants showed improvement in attitudes toward their own ability to manage their diabetes; in diet, physical activity, and foot care; and in hemoglobin A1c levels 3 months after the end of participation. Participants also reduced their sense of isolation when dealing with their diabetes. However, catering to the needs of a multiethnic community is extremely resource-intensive because of the need to provide adequate language and cultural interpretation.

**Conclusion:**

Self-management education can work in multiethnic, high-needs communities in New Zealand. Programs must ensure they enable the appropriate mechanisms and have appropriate resources to support the community's needs.

## Introduction

In New Zealand, the diabetes epidemic has captured public health officials' attention. The prevalence of diabetes is significantly higher in the South Auckland region compared with the rest of the country (1,2), and the population at risk for diabetes is growing. South Auckland also has a significantly higher prevalence of obesity in adults and children compared with national rates; 13% of children and 33% of adults are obese (2). This region is also one of the most ethnically diverse and deprived regions of the country; 17% of its population is Maori and 21% of its population Pacific (3). The Counties Manukau District Health Board (CMDHB) in South Auckland designed and promoted the Let's Beat Diabetes (4) self-management education (SME) primary health care program to help members of this culturally diverse community manage their diabetes.

Group-based training for self-management strategies for people with diabetes can be effective in improving fasting blood glucose, hemoglobin A1c (HbA1c), and diabetes knowledge (4). Further reviews reached similar conclusions (5-9) and led to a new paradigm of the doctor-patient relationship in chronic disease management, with SME as a key component (10). SME programs seek to help patients realize that they are their own principal caregivers and that health care professionals are consultants supporting patients in this role. Patients learn problem-solving skills and use action plans to find solutions to problems in the medical, social, and emotional aspects of their illness (11). To date, no published studies have investigated the cultural appropriateness or effectiveness of group SME with Pacific or Maori populations in New Zealand. Barriers to effective diabetes interventions with socially disadvantaged populations are language difficulties, cultural beliefs, lack of transportation, lack of time off work, lack of child care, low health literacy, and financial costs (12). Successful interventions need to recognize cultural differences and levels of literacy so that diabetes programs are relevant and accessible to their target population (13).

CMDHB commissioned an independent evaluation of the SME program, and this article presents the primary findings. The primary objective of the evaluation was to determine whether a 6-week group SME program would produce measurable results in participants' physical indicators, attitudes, and knowledge about diabetes management. We also sought to describe lessons learned from the implementation process and to learn whether the program met the diverse needs of a multicultural population.

## Methods

### Program description

The SME program was adapted from a similar program implemented in a neighboring district health board in 2006 to improve the uptake of best practices after a patient is diagnosed with diabetes. CMDHB worked through 5 of the 8 primary health organizations (PHOs) in Counties Manukau, which have direct patient care responsibilities, to recruit newly diagnosed diabetes patients. Each PHO recruited and employed a facilitator to lead 4 to 6 group patient education sessions of 2 hours each. The facilitator recruitment process varied among PHOs, as did area of expertise among recruits. Sessions were usually held in the evening, although a few morning groups were conducted for participants who had evening commitments. In addition, CMDHB hired a Maori facilitator and a Pacific facilitator who could speak Samoan and Tongan to provide added support for these populations. These facilitators acted as cultural interpreters and developed culturally appropriate variations of the SME program. For example, they incorporated singing and dancing in the Pacific program and used storytelling for the Maori program. The Maori SME sessions were held in a traditional Maori community house, called a marae. Other translators of several Pacific languages were also available to standard SME groups. PHOs agreed to collaborate across agencies throughout this program. One PHO hired a nutritionist as part of the facilitator team and collaborated with other PHOs to extend this expertise to other groups.

### Participant recruitment

General practitioners (GPs) or practice nurses in the PHO networks referred patients to the SME program. The program was intended for newly diagnosed patients, but because this was a new program, GPs referred any patient with diabetes who had been unable to develop good self-management skills and needed additional assistance. With the referral form, facilitators received baseline clinical data including measures of HbA1c, body mass index (BMI), and blood pressure. The SME facilitator contacted each patient to present the available and upcoming sessions and to help the patient solve logistical issues such as transportation. If the available program times suited the referred patient and the patient agreed to attend, the referred patient became an SME participant. SME programs ran continually from January 2007 until January 2008, and referrals were received throughout the study period.

### Program evaluation

All activities related to this evaluation were granted full ethical approval by the Ministry of Health's ethics committee. This evaluation used a mixed-methods approach. The study used 2 questionnaires to collect quantitative program data: a health attitude questionnaire and a health behavior questionnaire. The evaluation team in conjunction with other primary stakeholders developed and adjusted the questionnaires. The health attitude questionnaire consisted of a 1-page tool with items to be rated on a 5-point scale.

The study team adapted the tool presented by Toobert and colleagues (14) into a 1-page health behavior questionnaire with 14 items divided into 4 sections. We report results for 10 of the items in this article. Questions are, "In the last 7 days, how many days did you . . ." followed by a list of responses related to diet, physical activity, blood glucose testing, smoking frequency, medication, and foot care.

During the first session of each SME program, facilitators took 15 minutes at the beginning of the session to allow participants to respond to baseline questionnaires (pre). Participants then followed the SME program for 4 or 6 weekly sessions, and at the end of the last session completed the follow-up questionnaires (post). To obtain the 3-month follow-up information, facilitators organized a reunion at which participants completed the same 2 questionnaires for a third and last time. Both questionnaires were self-administered; therefore, facilitators did not assist participants unless they requested help because of a language or other limitation. For follow-up clinical data, participants' GPs provided HbA1c, BMI, and blood pressure measures with the patients' consent.

In addition, the evaluation team conducted 8 focus groups with program participants at the end of the last session of SME, ranging in duration from 45 to 80 minutes. The focus group facilitator invited all participants in attendance during the last session of SME to participate in the voluntary focus group. The objective of these focus groups was to obtain information about barriers and enablers to program development, implementation, and behavior change that could not be captured through the questionnaires. The evaluation team also conducted 12 interviews with SME facilitators and PHO managers, focusing on their perceptions of support and resources during program implementation and recommendations for further program development.

### Analysis

Quantitative data were analyzed by using SPSS version 15 (SPSS, Inc, Chicago, Illinois). Paired *t* tests were conducted to compare data from the health attitude and health behavior questionnaires through time; significance was set at *P* < .05. The analysis compared 1) participants' health attitudes and health behaviors pre-SME and post-SME (a 4- or 6-week difference between measures) < .05. The analysis compared 1) participants' health attitudes and health behaviors pre-SME and post-SME (a 4- or 6-week difference between measures) and 2) participants' health attitudes, health behaviors, and clinical outcome measures pre-SME and 3 months after program completion. The evaluation team conducted thematic analysis of the qualitative data by using NVivo version 8 (QSR International, Cambridge, Massachusetts).

## Results

A total of 193 people participated in an SME program during the study period. Participants' age ranged from 21 to 87 years; the mean (standard deviation) age was 57.6 (12.6) years. Two-thirds of the participants were women, and the 2 most commonly represented ethnicities were Pacific and Maori (40% and 37%, respectively) (Table 1).

Five of the 7 indicators of attitude improved significantly (Table 2). At 3-month follow-up, participants generally felt their health had improved, felt more confident about managing their diabetes, knew enough about diabetes to make choices that were right for them, felt good about living with diabetes, and had an improved understanding of diabetes.

Comparisons between the pre- and post-SME behavioral score show that there was a significant increase in the number of days participants reported eating at least 3 meals a day, eating breakfast, eating the recommended servings of fruits and vegetables, doing at least 30 minutes of moderate physical activity each day, and checking their feet (Table 3). The number of days participants reported eating high-fat foods decreased significantly.

However, when comparing the pre-SME scores with the 3-month follow-up behavioral scores (Table 4), the only noticeable sustained change was the decrease in the number of days participants reported eating high-fat foods. The only physical indicator that showed a significant improvement at 3-month follow-up was HbA1c, which was 8.0 compared with 8.4 (Table 5).

The focus group findings indicate that participants benefitted from the program. They reported feeling less isolated in their health condition and enjoying the social interaction that the group provided.

Coming to this course and listening to other people who have got the same situation, I've realized that it's not something that you have to suffer by yourself. [You can] talk to people that aren't obviously dying or anything and you just feel a bit more comfortable about the situation.

Language was highlighted as one of the main concerns for participants. Many preferred receiving information in their own language (mostly Pacific languages) and said that many community members would avoid participating in SME because of language concerns. Throughout the program, translators were made available when participants had language limitations that could not be addressed through facilitators. Despite this barrier, many participants indicated that they wanted to act as a bridge to close the gap between their communities and health services.

In our culture it's very hard for some of our people to acknowledge that they have these types of things [like diabetes]. So I want to be able to help the elders in my family as well to learn about it, to manage it, and to maintain a better health regime.

Participants revealed that facilitators were a highly motivated and committed group, who were familiar with the population and the local cultural context and were able to communicate well with their audience.

Well actually we've had nobody else like [facilitator]. He talked our language. We get other professionals coming in and using all these big words and we've got to go, "Hang on a minute." You know? But with [facilitator], we never ever said that because he spoke our language.

Facilitators reported several challenges to program implementation. Facilitators struggled to varying degrees to obtain data and had to negotiate with participants' GPs to gain access to some clinical information. Getting this information was often difficult, and together with post-program attrition explains the reduced sample size for statistical analyses. Participant recruitment also presented a challenge, particularly at the beginning of the program. Work schedules, transportation, and family commitments, as well as people's levels of readiness to engage in a health promotion program, affected levels of participation. Facilitators frequently said the target population was difficult to reach and named this a major barrier to change. Facilitators recognized stressful life circumstances and economic and family pressures as important determinants of participants' ability to change. Social and cultural environments were also identified as a barrier to participant learning, increasing the difficulty of implementing lifestyle changes that SME encourages.

These people are hard to reach . . . and they've got so many other stressors in their lives that for them to be ready to change and actually take control and be a good self-carer. . . . They've just [got] so much else on . . . whether they can pay the bills and have somewhere to live and feed the family and have a job.

Facilitators also reported that their organizations did not understand their role. Without exception, the SME facilitators were the first staff members to fulfill such a role in their organizations and therefore were mostly developing their role and establishing needs as the program was implemented. Facilitators reported that their managers often underestimated the amount of time required to prepare for SME sessions, leaving them feeling overworked. Lastly, for facilitators who were not implementing a culturally specific SME program, figuring out how to cater to participants' different language and cultural needs was a major challenge. Facilitators did not necessarily believe that segregating the groups by ethnicities was a good solution, pointing out that they live in a multiethnic community. Yet mastering the nutritional intricacies of several cultural groups and having language support added to the perception that this SME model was extremely resource-intensive.

## Discussion

Overall, the Let's Beat Diabetes program demonstrated significant changes in participant attitudes, some behavioral changes, and a small but statistically and clinically significant reduction in HbA1c levels. In addition to the measured achievements, valuable lessons were learned from the evaluation of the implementation process. From the beginning of program development, CMDHB and PHOs were willing to pool both human and organizational resources to support the implementation of SME. This type of interagency collaboration was unprecedented in this region and constitutes a success in itself. The program was developed assuming that an initiative built in the context of the community and facilitated by local providers would have a higher probability of success and sustainability because of the high level of cultural competency, which the focus group data confirmed.

Despite this success and the program's efforts to provide support and resources in many Pacific languages, language was perceived as a substantial barrier for many of the participants attending the SME programs that were not culturally specific. This perception highlights the importance of the language domain in SME. Attempting to engage with participants about psychosocial issues pertaining to health beliefs and behavior is a complex task that is further complicated by language barriers.

As with any program, organizational process can enable or hinder program process. SME facilitators experienced some challenges in the implementation of the program. Appropriate resources are necessary to success for any program; working with multiethnic groups is resource-intensive, and therefore ensuring the appropriate level of support for the facilitators' roles is imperative.

This project adopted program evaluation methods and was not a randomized clinical trial. This approach, combined with difficulties in obtaining follow-up clinical and questionnaire data, affected the overall sample size. Consequently, in-depth inferential statistical analysis was not feasible, which limited the study. Comparing the degree of success between the different cultural models of SME was also impossible because of lack of data.

Despite the lack of generalizability of study findings, this article presents data from the first coordinated effort at a community-based SME program in New Zealand dealing with a high-needs, multiethnic population. It provides an example of the degree of change that can be expected of interventions coordinated at a primary care level in these populations.

## Figures and Tables

**Table 1:** Characteristics of Participants (N = 193), Diabetes Self-Management Education Program, South Auckland, New Zealand, 2007-2008

**Characteristic^a^**	No. (%)
**Sex (n = 163)**	
Men	55 (34)
Women	108 (66)
**Age, y (n = 186)**	
≤45	30 (16)
46-55	48 (26)
56-65	59 (32)
≥66	49 (26)
**Ethnicity (n = 178)**	
New Zealand/European	10 (6)
Maori	66 (37)
Pacific	72 (40)
European	12 (7)
Indian	10 (6)
Other	8 (5)
**Primary health organization (n = 193)**	
East Tamaki Health Care	37 (19)
Procare	32 (17)
East Health	35 (18)
Mangere Community Health Trust	4 (2)
District Health Board – Pacific DSME	35 (18)
District Health Board – Maori DSME	50 (26)

Abbreviation: DSME, Diabetes Self-Management Education.

a Complete data were available for fewer than 193 participants for some categories because of program attrition and difficulty obtaining clinical data from participants' doctors.

**Table 2:** Attitude Scores of Participants (N = 193), Diabetes Self-Management Education Program, South Auckland, New Zealand, 2007-2008

Attitude Indicator^a^	Score, Mean (SD)		*P* Value^d^	Score, Mean (SD)	*P* Value^d^
	
	Baseline^b^ (n = 65)	Final^c^ (n = 65)		3-Month Follow-Up^e^ (n = 28)	
1. I think my health is . . .	2.8 (0.8)	3.4 (0.8)	<.001	3.8 (0.8)	.001
2. Managing my diabetes is mainly my responsibility.	4.5 (0.5)	4.7 (0.6)	.17	4.6 (0.5)	.80
3. I am motivated to care for my diabetes.	4.3 (0.7)	4.6 (0.5)	<.001	4.5 (0.8)	.26
4. I am confident that I can manage my diabetes.	4.1 (0.7)	4.4 (0.6)	<.001	4.7 (0.5)	.003
5. I know enough about diabetes to make choices that are right for me.	3.7 (0.9)	4.5 (0.5)	<.001	4.6 (0.5)	<.001
6. Most of the time I feel good about living with diabetes.	3.5 (1.1)	3.8 (1.1)	.07	4.3 (0.6)	.01
7. My understanding of diabetes and its management is . . .	2.9 (1.0)	4.1 (0.8)	<.001	4.2 (0.6)	<.001

Abbreviation: SD, standard deviation.

a Choices were 1 to 5; higher values represented more positive responses. For questions 1 and 7 choices were poor, fair, good, very good, or excellent. For questions 2 through 6 choices were strongly disagree, disagree, neither agree nor disagree, agree, or strongly agree.

b Data were collected at the beginning of the first session of the program. Complete data were available for fewer than 193 participants because of program attrition and difficulty obtaining clinical data from participants' doctors.

c Data were collected at the end of the last session, 4 or 6 weeks after collecting baseline data.

d Compared with baseline data; analyzed by using paired-sample *t* tests.

e Data were collected 3 months after the end of the self-management education program.

**Table 3:** Behavioral Scores of Participants (N = 193) at Last Program Session, Diabetes Self-Management Education Program, South Auckland, New Zealand, 2007-2008

Activity	n	No. of Days^a^ Doing Activity, Mean (SD)		*P* Value^d^

		Baseline^b^	Final^c^	
Eating at least 3 meals	71	5.4 (2.1)	6.1 (1.4)	.001
Eating breakfast	71	5.9 (1.9)	6.4 (1.4)	.004
Eating at least 2 servings of fruit	69	4.9 (2.3)	5.8 (1.5)	.002
Eating at least 3 servings of vegetables	70	5.3 (1.9)	5.8 (1.4)	.04
Eating high-fat foods	68	2.6 (1.9)	1.5 (1.5)	<.001
Doing at least 30 minutes of moderate activity	71	4.7 (2.3)	5.3 (1.9)	.01
Doing planned exercise sessions	66	3.0 (2.4)	3.5 (2.5)	.16
Testing blood glucose	49	3.6 (2.5)	4.2 (1.9)	.14
Taking recommended diabetes medicine	55	6.6 (1.5)	6.6 (1.3)	.76
Checking feet	66	3.4 (3.1)	4.4 (2.6)	.006

Abbreviation: SD, standard deviation.

a In previous 7 days.

b Data were collected at the beginning of the first session of the program. Complete data were available for fewer than 193 participants because of program attrition and difficulty obtaining clinical data from participants' doctors.

c Data were collected at the end of the last session, 4 or 6 weeks after collection of baseline data.

d Paired-sample *t* tests were used to analyze the difference between baseline and follow-up measures.

**Table 4:** Behavioral Scores of Participants (N = 193) at 3-Month Follow-Up, Diabetes Self-Management Education Program, South Auckland, New Zealand, 2007-2008

Activity	n	No. of Days^a^ Doing Activity, Mean (SD)		*P* Value^d^

		Baseline^b^	3-Month Follow-Up^c^	
Eating at least 3 meals a day	28	5.7 (1.5)	6.0 (1.6)	.39
Eating breakfast	28	5.6 (2.1)	6.3 (1.5)	.08
Eating at least 2 servings of fruit	28	4.6 (2.3)	5.3 (1.6)	.13
Eating at least 3 servings of vegetables	28	4.9 (1.9)	5.6 (1.6)	.12
Eating high-fat foods	28	3.1 (2.2)	1.5 (1.0)	.001
Doing at least 30 minutes of moderate activity	28	5.0 (2.4)	5.7 (1.9)	.20
Doing planned exercise sessions	28	2.8 (2.6)	3.4 (2.2)	.22
Testing blood glucose	14	2.9 (2.3)	2.9 (2.5)	>.99
Taking recommended diabetes medicine	15	6.0 (2.0)	6.2 (2.2)	.46
Checking feet	27	2.2 (2.9)	3.0 (2.4)	.10

Abbreviation: SD, standard deviation.

a In previous 7 days.

b Data were collected at the beginning of the first session of the program. Complete data were available for fewer than 193 participants because of program attrition and difficulty obtaining clinical data from participants' doctors.

c Data were collected 3 months after the last program.

d Paired-sample *t* tests were used to analyze the difference between baseline and follow-up measures.

**Table 5:** Health Indicator Outcomes of Participants (N = 193), Diabetes Self-Management Education Program, South Auckland, New Zealand, 2007-2008

Indicator	n	Mean (SD)		*P* Value^c^

		Baseline^a^	3-Month Follow-Up^b^	
HbA1c, %	48	8.4 (1.8)	8.0 (2.0)	.04
BMI, kg/m^2^	40	31.8 (8.1)	31.7 (8.1)	.95
Systolic blood pressure, mm Hg	51	131.3 (17.0)	127.5 (18.9)	.09
Diastolic blood pressure, mm Hg	51	78.8 (1.8)	76.2 (11.2)	.10

Abbreviations: SD, standard deviation; HbA1c, hemoglobin A1c; BMI, body mass index.

a Data were collected at the beginning of the first session of the program. Complete data were available for fewer than 193 participants because of program attrition and difficulty obtaining clinical data from participants' doctors.

b Data were collected 3 months after the end of the program.

c Paired-sample *t* tests were used to analyze the difference between baseline and follow-up measures.
